# Patterns of Lesbian, Gay, Bisexual, Transgender, and Queer Patient Experiences and Receipt of Preventive Services

**DOI:** 10.1111/1475-6773.14632

**Published:** 2025-05-04

**Authors:** Nathaniel M. Tran, Gilbert Gonzales, Carrie E. Fry, Stacie B. Dusetzina, Tara McKay

**Affiliations:** ^1^ Division of Health Policy and Administration University of Illinois Chicago School of Public Health Chicago Illinois USA; ^2^ Department of Health Policy Vanderbilt University Medical Center Nashville Tennessee USA; ^3^ Department of Medicine, Health, and Society Vanderbilt University Nashville Tennessee USA

**Keywords:** clinical competency, cultural competency, LGBTQ+, patient experience, preventive services

## Abstract

**Objective:**

To identify patterns of LGBTQ+ patient experiences, to identify sociodemographic characteristics associated with patterns of LGBTQ+ patient experiences, and to assess the relationship between LGBTQ+ patient experience and receipt of preventive services.

**Study Setting and Design:**

This observational cohort study included adults across the U.S. South. We conducted latent class analysis of seven indicators of clinical and cultural competency to identify patterns of LGBTQ+ patient experiences. Outcomes included the proportion of respondents with lifetime and recent influenza vaccination, HIV testing, and colorectal cancer screening.

**Data Sources and Analytic Sample:**

Data come from Waves 1 and 2 of the LGBTQ+ Social Networks, Aging, and Policy Study collected between April 2020 and October 2022. The sample included 954 LGBTQ+ adults ages 50–76 living in Tennessee, Georgia, Alabama, or North Carolina at baseline.

**Principal Findings:**

We identified three patterns of LGBTQ+ patient experiences. 34% of the sample reported LGBTQ+ affirming care, 60% reported neutral care, and 6% reported discriminatory care. Gender identity, sexual orientation, race and ethnicity, state of residence, and HIV status predicted patterns of patient experiences (all *p* < 0.01). Compared to patients with affirming care, patients with neutral care were 12.4 percentage points less likely to have ever been tested for HIV (*p* < 0.0001) and 17.1 percentage points less likely to have been recently tested for HIV (*p* < 0.0001); patients reporting discriminatory care were 12.2 percentage points less likely to have recently received an influenza vaccination (*p* = 0.024) and 14.8 percentage points less likely to have recently completed a colorectal cancer screening (*p* = 0.035).

**Conclusions:**

In the absence of explicitly LGBTQ+ affirming patient experiences, LGBTQ+ midlife and older adults are less likely to receive preventive services such as colorectal cancer screenings, influenza vaccinations, and HIV testing. Interventions to increase the capacity of health systems to provide LGBTQ+ affirming care are needed to advance health equity.

**Summary**

- What is known on this topic
  ○ Lesbian, gay, bisexual, transgender, and queer (LGBTQ+) patients commonly report healthcare discrimination and underutilization of preventive services.
  ○ Racial and sex concordance between patients and clinicians has been shown to improve patient experiences, increase uptake of preventive services, and reduce long‐term mortality.
- What this study addss
  ○ LGBTQ+ clinical and cultural competency can be measured when clinician identity is not available to study LGBTQ+ patient experiences.
  ○ LGBTQ+ patient experiences vary widely. One third (34%) of LGBTQ+ patients reported affirming care, 60% reported neutral care, and 6% reported discriminatory care.
  ○ LGBTQ+ affirming patterns of patient experiences were associated with the highest levels of preventive services such as influenza vaccination, colorectal cancer screening, and HIV testing.

## 1 Introduction

Healthcare‐based discrimination erodes trust, reduces engagement with health systems, and remains an acute challenge for LGBTQ+ (i.e., lesbian, gay, bisexual, transgender, and queer/questioning) populations [1, 2]. Section 1557 of the Affordable Care Act includes non‐discrimination protections for federally protected classes, including sex. In May 2016, the Obama administration issued a proposed rule explicitly interpreting sex stereotype protections to include sexual orientation and gender identity [3]. As recent as November 2022, the U.S. District Court of North Texas ruled that healthcare providers “need not comply with the interpretation of ‘sex’ discrimination” that includes sexual orientation and gender identity [4]. In the absence of federal non‐discrimination protections, state‐level policies provide a patchwork of legal protections from healthcare discrimination on the basis of LGBTQ+ identity [5].

Meanwhile, LGBTQ+ midlife and older adults experience substantial health and healthcare inequities [6]. Despite a higher risk for multiple cancers and chronic conditions, LGBTQ+ adults are less likely to receive necessary preventive screenings [7, 8, 9, 10]. Compared to non‐LGBTQ+ populations, LGBTQ+ adults are also less likely to be offered and receive preventive services such as screening for sexually transmitted infections or vaccinations for conditions like hepatitis or human papillomavirus [11, 12, 13, 14]. Negative healthcare experiences, including not being treated with respect, being asked invasive questions, or being told to seek care elsewhere, further contribute to avoidance and delays in seeking care among LGBTQ+ populations. Moreover, LGBTQ+ health disparities are greater in the southern United States (where public policy is less LGBTQ+ affirming) compared to other regions of the country and are partially attributable to fewer healthcare resources tailored to LGBTQ+ populations and fewer healthcare resources overall [15, 16].

Strong patient‐clinician therapeutic relationships help patients make better decisions about what type of preventive services to receive [17, 18], identify treatment options that align with their treatment preferences, and better manage chronic conditions [19]. One approach to strengthening therapeutic relationships is studying how outcomes differ when patients are matched to their provider on characteristics such as race or gender. Prior studies posit that patient and clinician identity concordance should reduce bias towards the treatment of minoritized patients. In observational studies, racial concordance was associated with higher participatory decision‐making and higher patient satisfaction scores, which are expected to improve the quality of care [20, 21]. Additional experimental evidence finds racial and gender concordance increases the probability of accurate disability evaluations, improves the uptake of preventive services, and lowers cardiovascular mortality [22, 23, 24]. These studies suggest that diversity in the workforce, particularly by matching patients to their clinician on racial or gender identity characteristics, may improve the therapeutic relationship and, in turn, improve the receipt of preventive services and the health of patients.

To date, no studies have tested the relationship of LGBTQ+ patient and clinician identity concordance. This research question remains challenging as no large‐scale datasets assess both patient and clinician sexual orientation or gender identity [25, 26, 27]. Instead, researchers might examine health outcomes among LGBTQ+ patients with clinicians who actively affirm the identity and healthcare needs of their LGBTQ+ patients. In the current study, we apply a previously established multi‐level conceptual model of LGBTQ+ affirming care [28]. At the interpersonal level, affirming an LGBTQ+ patient may be composed of both cultural and clinical competence. Cultural competence refers to a provider's preparedness to treat LGBTQ+ people in a safe and respectful manner, which might include using inclusive language on forms and in person (e.g., *What are your pronouns? Are you partnered? How does your partner identify?)*. Clinical competence refers to having the scientific and translational knowledge of LGBTQ+ health needs to provide high‐quality, evidence‐based treatment, which may be developed through didactic lectures or continuing education (e.g., *Have you had opportunities to counsel and manage patients who may be good candidates for HIV preexposure prophylaxis (PrEP)?)* The multi‐level conceptual model also includes additional institutional‐level and structural‐level factors that are beyond the scope of this study.

Using data from the Aging with Pride study (*n* = 2450) and latent class analysis, researchers investigated LGBTQ+ patient help seeking behaviors. This study used patient‐level factors such as health behaviors and healthcare barriers to generate latent classes and to assess the relationship between class membership and health‐related quality of life. Specific healthcare barriers included financial barriers to care, availability of LGBTQ+ friendly services, distrust in healthcare, health literacy, postponement of care, and willingness to receive care. Researchers found that LGBTQ+ patients with high rates of healthy behaviors and low barriers to healthcare had the best health‐related quality of life, whereas LGBTQ+ patients with less healthy behaviors and barriers to healthcare had poorer physical quality of life; LGBTQ+ patients with healthy behaviors and barriers to healthcare had poorer psychological quality of life [29]. This study focused on patient‐reported experiences in healthcare rather than clinician factors that inform utilization of care and demonstrates the utility of latent class approaches to studying variation in health within LGBTQ+ populations.

Using baseline data from the longitudinal LGBTQ+ Social Network, Aging, and Policy Study (Q‐SNAPS), researchers found that older LGBTQ+ adults who reported that their clinician was LGBTQ+ affirming were more likely to receive recommended vaccinations, HIV testing, and colorectal cancer screening, were more likely to report better management of their chronic mental health conditions, and to report better understanding and belief in “*undetectable = untransmittable*” HIV prevention messaging used by the Centers for Disease Control and Prevention (CDC) [28, 30]. This analysis of Q‐SNAPS data used a binary measure comparing respondents with an affirming provider to those who did not want, need, or could not find an affirming provider. LGBTQ+ patient experiences vary widely and likely do not neatly collapse into a single indicator and may be complex or even counterintuitive. For example, some LGBTQ+ patients may receive care from explicitly affirming providers, while others may not have an affirming provider. We hypothesize that a subset of patients who do not have an affirming provider belong to a “neutral” class without negative or positive experiences. Such neutral providers might be ideal candidates for receiving clinical and cultural competency training.

The objectives of this study are to (1) identify patterns (latent classes) of LGBTQ+ patient experiences using seven indicators of clinical and cultural competency, (2) identify sociodemographic characteristics associated with patterns of care, and (3) assess the relationship between LGBTQ+ patient experiences and receipt of influenza vaccinations, colorectal cancer screenings, and HIV testing.

## 2 Methods

### 2.1 Data and Eligibility

This observational cohort study followed STROBE reporting guidelines and used longitudinal data from Q‐SNAPS, a longitudinal study of LGBTQ+ adults aged 50–76 years living in Tennessee, Georgia, Alabama, and North Carolina. Wave I data (*n* = 1256) were collected between April 2020 and September 2021, and Wave II data (*n* = 1138) were collected between December 2021 and September 2022. Participants were recruited through outreach to organizations and at events serving LGBTQ+ communities and older adults, as well as via paid advertisements on social media platforms. Among 1138 respondents available for follow‐up at Wave II, 69 skipped the clinical and cultural competency measures; 42 preferred not to answer one or more of the clinical and cultural competency indicators; and 73 did not have a healthcare appointment since the last survey or preferred not to answer one of the indicators. The final analytic sample included 954 respondents with linked Wave I and Wave II data who met eligibility criteria. At baseline, respondents self‐reported demographic characteristics including sexual orientation, gender identity, race, and ethnicity. Response options included Asian, Black or African American, Hispanic or Latino, Native Indian or Alaskan Native, White, and an open response category not another race or ethnicity not provided.

### 2.2 Measures

#### 2.2.1 Clinical and Cultural Competence Measure

Wave 2 of Q‐SNAPS included a 7‐item patient‐reported measure of their providers' clinical and cultural competence, or lack thereof, for working with LGBTQ+ patients. This measure was developed as part of a community‐engaged research drawing on interviews and cross‐sectional survey data from a single large city [31], however, this measure has not yet been formally rated.

Participants were asked “*Thinking about the time since the last survey [Wave I], about how often did you experience each of the following?*” about the following experiences: My doctor or other healthcare provider had materials in their office that let me know they were LGBTQ+ friendly, My doctor or other healthcare provider used inclusive language in‐person or on forms that let me know they were LGBTQ+ friendly, I had to teach my doctor or other healthcare provider about LGBTQ+ people so that I could get appropriate care, A doctor or other healthcare provider advised me to seek care elsewhere because they did not have knowledge or certifications to provide me with proper care, I left my healthcare appointment feeling like all my questions or concerns had been addressed, My doctor or other healthcare provider asked me unnecessary/invasive questions about my LGBTQ+ status that were not related to the reason for my visit, and I felt comfortable asking my doctor or other healthcare provider about all aspects of my health or care. Potential response options included: *Always*, *Often*, *Sometimes*, *Rarely*, *Never*, *Not applicable, I did not have any healthcare appointments since the last survey*, or *I prefer not to answer*. Respondents who reported *I did not have any healthcare appointments since the last survey* or *I prefer not to answer* were ineligible for the current study. Responses *Always*, *Often, Sometimes* were dichotomized as “Yes,” and responses *Rarely* and *Never* were dichotomized as “No.” When data are not normally distributed, binarizing indicator variables may produce a less biased estimator in structural equation modeling techniques [32]. (Figure S1 shows the distribution of responses to the original indicators.)

#### 2.2.2 Preventive Service Outcome Measures

We leveraged Q‐SNAPS' longitudinal panel design to link Wave I and Wave II using a unique participant identification key to assess receipt of preventive services. At Wave I, respondents were asked “*Have you ever had any of the following preventive care screenings or tests?*” including influenza vaccination, colorectal cancer screening or colonoscopy, and HIV testing which was coded as ever received. At Wave II, all participants were asked “*Which of these tests or screenings have you had in the last 3 years?*” which was coded as recently received. All preventive service outcomes in this study are recommended for the full Q‐SNAPS sample based on recommendations from the US Preventive Task Force.

### 2.3 Statistical Analysis

We used latent class analysis, which provides a data‐driven approach for fitting observable characteristics (indicators) into indirectly measured (latent) sub‐groups. We used seven indicator variables to identify potential latent classes of LGBTQ+ patient experiences. Rather than measuring binary indicators of LGBTQ+ healthcare experiences (affirming or non‐affirming), we hypothesize that a subset of respondents experiences more complex patterns of experience (e.g., both explicitly affirming and discriminatory experiences with their clinician in a single visit or neither explicitly affirming or discriminatory experiences with their clinician).

Identifying the appropriate number of latent classes relies on multiple statistical and theoretical approaches. One approach is to use statistical measures of goodness of fit such as Akaike information criterion (AIC) or Bayesian information criterion (BIC), both of which assess how well a statistical model fits the observed data as well as the proposed complexity of the model (increasing number of classes). A second type of statistical measure of goodness of fit is the Lo–Mendell–Rubin likelihood ratio test, which systematically tests whether the null hypothesis that a model of *k* number of classes outperforms a model with *k*‐1 number of classes. Parsimony is an additional theoretical approach for identifying the appropriate number of latent classes by assessing the complexity of the latent class analysis model. Parsimony is especially helpful when goodness of fit statistics do not identify the same model that optimally fits the data. In this study, we estimate models with one to five classes and assess model performance using goodness of fit statistics [33, 34, 35] and parsimony, guided by qualitative findings on experiences of and responses to healthcare discrimination among LGBTQ+ and other historically marginalized populations [36, 37, 38, 39].

After identifying the best fitting model, respondents are assigned to the class with the highest posterior probability. We then test the distribution of patient sociodemographic characteristics and class membership using Pearson's chi‐squared test. Lastly, we estimate the unadjusted marginal effects for each latent class on the receipt of preventive services using logistic regression to assess whether and how latent class assignment is associated with the receipt of recommended preventive healthcare. All analyses were conducted using Stata v17.0. This study was deemed non‐human subjects research by the Vanderbilt University Institutional Review Board.

## 3 Results

### 3.1 Goodness of Fit of Latent Class Models

We first assessed goodness of fit using the AIC and BIC values and likelihood ratio test. Taken together, the 3‐class model has the best performance for the data. Using a 3‐class model, respondents were assigned to the class with the highest posterior probability, with 34% of the sample assigned to the Affirming class, 60% to the Neutral class, and 6% to the Discriminatory class.

Table 1 presents full goodness of fit statistics and class distribution of latent class models. The 3‐class model outperforms the 2‐class model by reducing AIC and slightly increasing BIC (which penalizes more complex models), and the 4‐class model outperforms the 3‐class model by reducing both AIC and BIC. The 5‐class model fails to outperform the 4‐class model by increasing BIC. Next, we compare the likelihood ratio test. The 2‐class model outperforms a 1‐class model (*p* < 0.0001), and a 3‐class model outperforms a 2‐class model (*p* < 0.0001). A 4‐class model fails to outperform the 3‐class model (*p* = 0.908) and a 5‐class model fails to outperform a 4‐class model (*p* = 0.983). In addition to goodness of fit statistics, we also select the best fitting latent class models using parsimony. The 4‐class model and 5‐class model both generate a class with less than 2% of the total sample; therefore, they fail to outperform the 3‐class model due to insufficient class size.

**TABLE 1:** Fit statistics and distribution of LGBTQ+ patient experiences across latent classes.

	2‐Class	3‐Class	4‐Class	5‐Class
Panel A: Goodness of fit statistics				
Akaike's information criterion (AIC)	4983.05	4945.18	4856.77	4856.60
Bayesian information criterion (BIC)	5055.96	5056.97	4997.73	5036.45
Likelihood ratio test (*p* value)	< 0.0001	< 0.0001	0.908	0.983
Panel B: Class membership				
Class #1	63%	60%	58%	50%
Class #2	37%	34%	27%	24%
Class #3	—	6%	13%	12%
Class #4	—	—	2%	12%
Class #5	—	—	—	2%

In the overall sample, 34% of respondents reported LGBTQ+ friendly materials in the clinic; 61% reported inclusive language in person and on paperwork; 92% reported comfort discussing all aspects of their care; and 77% reported feeling like their concerns were addressed. Regarding negative healthcare experiences, 3% of the combined sample reported being asked invasive questions about their LGBTQ+ identity; 5% reported being told to seek care elsewhere; and 8% reported having to teach their clinician about LGBTQ+ people to get adequate care.

**FIGURE 1:**
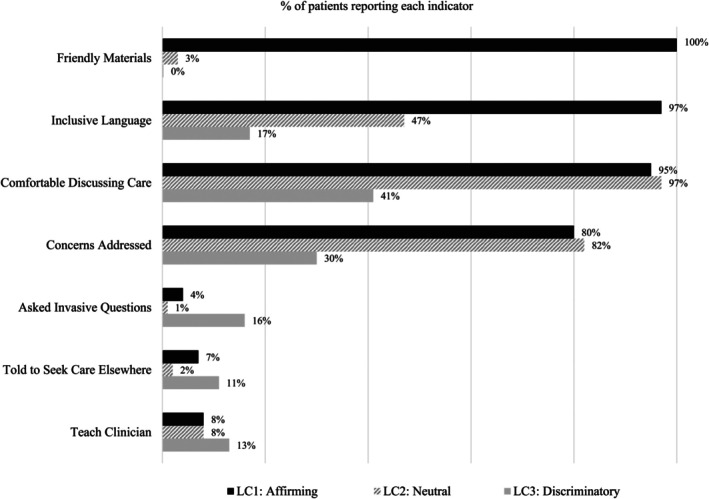
Distribution of LGBTQ+ patient experience indicators for 3‐class model.

Figure 1 presents the class‐specific distribution of responses to the seven indicator variables included in the latent class analysis. Of the patterns of LGBTQ+ patient experiences, affirming care (34% of the sample) represents LGBTQ+ patients reporting high affirmation, moderately high patient satisfaction, and low discrimination. Neutral care (60% of the sample) represents LGBTQ+ patients reporting low affirmation, moderately high patient satisfaction, and low discrimination. Lastly, discriminatory care (6% of the sample) represents LGBTQ+ patients distinguished by low affirmation, low patient satisfaction, and high discrimination.

Affirming care (34% of the sample) represents LGBTQ+ patients distinguished by high affirmation, moderately high patient satisfaction, and low discrimination. This class has a 100% rate of LGBTQ+ friendly materials in the clinic and a 97% rate of inclusive language in person and on paperwork; a 95% rate of comfort discussing all aspects of their care and an 80% rate of feeling like their concerns were addressed; a 4% rate of being asked invasive questions, a 7% rate of being told to seek care elsewhere, and an 8% rate of having to teach their clinician about LGBTQ+ appropriate care.

Neutral care (60% of the sample) represents LGBTQ+ patients reporting low affirmation, moderately high patient satisfaction, and low discrimination. This class has a 3% rate of LGBTQ+ friendly materials in the clinic and a 47% rate of inclusive language in person and on paperwork; a 97% rate of comfort discussing all aspects of their care and an 82% rate of thinking their concerns were addressed; a 1% rate of being asked invasive questions, a 2% rate of being told to seek care elsewhere, and an 8% rate of having to teach their clinician about LGBTQ+ appropriate care.

Discriminatory care (6% of the sample) represents LGBTQ+ patients distinguished by low affirmation, low patient satisfaction, and high discrimination. This class has a 0% rate of LGBTQ+ friendly materials in the clinic and 17% rates of inclusive language in person and on paperwork; a 41% rate of comfort discussing all aspects of their care and a 30% rate of thinking their concerns were addressed; a 16% rate of being asked invasive questions, an 11% rate of being told to seek care elsewhere, and a 13% rate of having to teach their clinician about LGBTQ+ appropriate care.

### 3.2 Sociodemographic Predictors of Class Membership

Table 2 presents the distribution of sociodemographic characteristics by pattern of LGBTQ+ patient experience. Overall, gender identity, sexual orientation, race and ethnicity, state of residence, and HIV status were significant predictors of class membership (all *p* < 0.01), but not education level, household income, health insurance status, or having chronic conditions (Table 3). Transgender and gender diverse respondents, Black and Hispanic respondents, respondents living in Georgia and North Carolina, and people living with HIV were most likely to report affirming care. Cisgender women, bisexual respondents, White respondents, and respondents from Alabama were more likely to report neutral care. Non‐LGB respondents and respondents living in Tennessee were most likely to report discriminatory care.

**TABLE 2:** Sociodemographic characteristics associated with patterns of LGBTQ+ patient experiences.

	Class 1: Affirming care	Class 2: Neutral care	Class 3: discriminatory care	*p*
Gender identity				< 0.001
Cisgender man	40.5	53.6	5.9	
Cisgender woman	22.4	71.6	6.0	
Transgender and gender diverse	47.3	45.5	7.3	
Sexual orientation				< 0.001
Gay/lesbian	34.4	59.8	5.8	
Bisexual	24	68.8	7.3	
Not LGB	44.4	37	18.5	
Race and ethnicity				0.01
Black or African American	41.1	51.8	5.2	
Hispanic or Latino	42.1	47.4	5.4	
Other	30.6	50.0	6.3	
White	32.9	61.7	8.9	
Education				0.15
Less than college	32.6	58.5	8.9	
College degree	37.3	57.5	5.2	
Grad/professional degree	31.5	63.1	5.4	
Household income				0.31
< 45 k	37.3	56.1	6.6	
45–75 k	32.4	63.4	4.2	
75–120 k	35.5	58.5	6	
125 k+	29.3	62.2	8.4	
State				0.001
Alabama	27.6	67.2	5.2	
North Carolina	35.2	59.6	5.2	
Tennessee	27.6	63.6	8.8	
Georgia	45.1	49.8	5.2	
Health insurance				0.11
Yes	33.5	60.6	5.9	
Chronic conditions				0.14
1+	33.9	60.3	5.8	
HIV status				< 0.001
Positive	76.5	20.6	2.9	

*Note*: Other race and ethnicity included Asian, Native Indian or Alaskan Native, and open response category for a race and ethnicity not provided.

**TABLE 3:** Marginal effects of pattern of LGBTQ+ patient experience on receipt of preventive services.

	Overall (%)	Affirming (%)	Neutral (%)	Affirming – Neutral (∆)	Discriminatory (%)	Affirming – Discriminatory (∆)
Influenza vaccination, ever	89.1	90.0	88.3	−1.7	91.7	1.6
*p*‐value				0.422		0.678
HIV test, ever	61.6	69.8	57.4	−**12.4**	58.3	−11.5
*p*‐value				**<0.0001**		0.095
CRC screening, ever	78.8	81.0	78.4	−2.6	71.7	−9.3
*p*‐value				0.344		0.134
Influenza vaccination, recent	89.9	92.2	89.6	−2.6	80.0	−**12.2**
*p*‐value				0.194		**0.024**
HIV test, recent	29.2	39.8	22.8	−**17.1**	33.3	−6.5
*p*‐value				**<0.0001**		0.332
CRC screening, recent	57.5	61.4	56.4	−5.0	46.7	−**14.8**
*p*‐value				0.145		**0.035**

*Note*: Marginal effects estimated using logistic regression. ∆ = difference between two groups. Decimals may vary by one‐tenth decimal place due to rounding. Bolded values are point estimates that are statistically significant at the 0.05 level.

Abbreviation: CRC = colorectal cancer screening.

### 3.3 Marginal Effects of Pattern of LGBTQ+ Patient Experience on Receipt of Preventive Services

We estimate the marginal effects of latent class membership on receipt of all preventive services measures assessed. Table 3 presents full regression estimates of the marginal effects of latent class pattern of LGBTQ+ patient experience and receipt of preventive services. Compared to individuals reporting affirming care, those with neutral care were 12.4 percentage points (*p* < 0.0001) less likely to have ever been tested for HIV and 17.1 percentage points (*p* < 0.0001) less likely to have been recently tested for HIV. Compared to individuals assigned to affirming care, those with discriminatory care were 12.2 percentage points (*p* = 0.024) less likely to have recently received a flu vaccination and 14.8 percentage points (*p* = 0.035) less likely to have recently completed a colorectal cancer screening.

## 4 Discussion

The present study used latent class analysis to describe patterns of LGBTQ+ patient experiences among a cohort of LGBTQ+ adults aged 50–76 years in the southern United States. In the overall sample, 34% of respondents reported LGBTQ+ friendly materials offered in the clinic, and 61% reported inclusive language in person and on paperwork. Regarding negative healthcare experiences, 3% of patients reported being asked invasive questions about their LGBTQ+ identity, 5% reported being told to seek care elsewhere, and 8% reported having to teach their clinician about appropriate care for LGBTQ+ people. Healthcare experiences within the sample varied widely, and latent class analysis results suggest that patients had three distinct patterns of LGBTQ+ patient experiences: (1) 34% of the sample had affirming care marked by high rates of positive experiences and low rates of negative experiences; (2) 60% of the sample had neutral care with low rates of positive and negative experiences; and (3) 6% of the sample had discriminatory care with low rates of positive experiences and high rates of negative experiences. In the absence of explicitly affirming care, LGBTQ+ patients reporting neutral or discriminatory care reported lower rates of 1 or more US Preventive Task Force recommended preventive services.

By sample construction (at least one healthcare appointment since Wave I) the transgender and gender diverse respondents and respondents living with HIV likely represent patients who have successfully identified and established a therapeutic alliance with an affirming provider. Respondents living in Georgia and North Carolina were more likely to report affirming patterns of patient experiences, which may reflect institution‐level predictors of access to affirming care with more healthcare infrastructure. Drawing on supplemental data from the Human Rights Campaign's Healthcare Equality Index, we find that 11 health systems in Georgia and 32 in North Carolina were rated as top performers or leaders in LGBTQ+ health equity, compared to 5 in Tennessee and 4 in Alabama (See Table S1 presents full Healthcare Equality Index data for healthcare systems in Q‐SNAPS sample states.) Considering the low number of health systems with adequate capacity for serving LGBTQ+ patients and our finding that 60% of LGBTQ+ patients reported neutral patterns of experiences—interventions such as LGBTQ+ focused clinical curricula and continuing education should be implemented to increase system‐level capacity for serving LGBTQ+ patients.

Class membership significantly predicted unique differences in suboptimal receipt of preventive services. Compared to respondents with affirming care, respondents with neutral care were less likely to report lifetime or recent HIV testing. One explanation of this finding is that patients with neutral care include a larger share of cisgender women who may be missed by historical recommendations for HIV testing, which have historically focused on gay and bisexual men. The US Preventive Task Force recommends that all adolescents and adults aged 15–65 years be screened for HIV at least once, regardless of sexual orientation and/or gender identity. Outside of this age range, populations at higher risk for HIV infection (including adults older than 65) should continue to be screened. Key risk factors include condomless anal intercourse, condomless vaginal intercourse with 1 or more partners whose HIV status is unknown, and individuals who recently had sex with a partner who has recently been diagnosed with a sexually transmitted infection. Low prevalence of lifetime and recent HIV testing is of high concern, given that 19% of new HIV diagnoses occur among heterosexual contacts among females [40], and rates of new HIV infection have not declined for cohorts aged 55 years and older since 2017 [41].

Compared to respondents with affirming care, respondents with discriminatory care were less likely to have recently received a flu vaccination or a colorectal cancer screening. Interventions that reduce disparities in the receipt of preventive services in minoritized populations, including LGBTQ+ populations, reduce the overall strain on health and social resources (e.g., healthcare expenditures and short term disability) for the general population. When clinicians discriminate against LGBTQ+ people, not only do LGBTQ+ people avoid receiving preventive services (e.g., colorectal cancer screening) but society bears the burden of costly preventable disease (e.g., colorectal cancer).

This study is subject to several limitations. Patient‐centered reports of clinical and cultural competence were assessed only during the period between Wave I and Wave II. This lookback period may not fully capture the relationship between lifetime experiences of healthcare discrimination, affirming patient–clinician relationships, and preventive service outcomes. Patients who experience severe discrimination prior to Wave I may have avoided care during the period between Wave I and Wave II, thus being ineligible for the present study. A second limitation is that the study sample is from the southern US and may not be representative of healthcare experiences of LGBTQ+ adults in other US regions. Despite this limitation, Q‐SNAPS remains the best data source available to address the proposed research question, given that it is the largest sample of LGBTQ+ adults in midlife and older age in the US with detailed information about clinical and cultural competency. Receipt of preventive services is self‐reported and may be subject to recall bias, leading to underestimates of actual receipt. However, the potential of recall bias for HIV testing and colorectal cancer screening is low due to the invasive nature (e.g., blood draw, colonoscopy) of these procedures. Receipt of HIV testing may be underreported as testing no longer requires consent and may be included with other labs conducted during routine primary care visits. Lastly, our measure of provider LGBTQ+ competency has not been previously validated. Existing validated measures of LGBTQ+ competency focus on pre‐licensure nursing curriculum [42] and social work practice [43], but no measure has focused on primary care clinicians' LGBTQ+ competency. Research that develops, tests, and validates an LGBTQ+ competency measure would advance the field of health services research.

Our results underscore the benefits of affirming healthcare experiences for LGBTQ+ patients, including timely receipt of preventive services. Using a large, multi‐state sample of LGBTQ+ patients, we find that the minority of clinicians (5%) told LGBTQ+ patients to seek care elsewhere. Ongoing litigation asks whether or not clinicians can legally invoke conscientious objections to refuse care to LGBTQ+ patients if doing so constitutes a conflict of their religious or closely held beliefs [44]. More commonly, clinicians report that their training at the undergraduate, graduate, and postgraduate levels did not include adequate structured learning about clinical and cultural approaches to caring for LGBTQ+ patients [45, 46, 47, 48, 49, 50, 51, 52]. Increasing the capacity of clinicians to establish affirming therapeutic relationships with LGBTQ+ patients via LGBTQ+ inclusive clinical education may be an important, modifiable pathway to promote LGBTQ+ health equity.

At the department level, clinical leaders should engage in conversations about barriers and facilitators of adopting LGBTQ+ competencies. For example, the collection of sexual orientation and gender identity data in electronic health records is technologically feasible (fields can be added), but requests require buy‐in from administrators and clinicians. Large gaps remain in the collection of sexual orientation and gender identity information within electronic health records, posing a challenge to achieving LGBTQ+ health equity [53].

At the institutional level, health system leaders should engage in systematic and sustained efforts to champion LGBTQ+ health equity. One framework for doing so is participation in the Human Rights Campaign's Healthcare Equality Index (HEI). The HEI is a structured toolkit for health systems to self‐assess the equity implications of policies and practices towards LGBTQ+ patients and staff. Sample performance goals of aligning policies and practices to advance LGBTQ+ health equity might include: “A*re same‐sex partners who are not legally married permitted equal visitation rights as opposite‐sex partners?*” and “*Does at least one health plan offered cover gender affirming healthcare services?*” The HEI designates health systems that meet required and optional performance goals as LGBTQ+ health equity leaders. These tiers of performance incentivize health systems to conduct a baseline evaluation of their policies and practices, as well as offer a stepwise path towards improving their performance. Previous research finds that supportive hospital‐level policies and practices towards LGBTQ+ populations was associated with higher patient satisfaction, but studies were not able to identify differences by sexual orientation or gender identity [54]. Findings from this study suggest that respondents in states with more HEI participating health systems may be more likely to be report clinical practices that affirm LGBTQ+ patients.

The Health Resources and Services Administration and the Joint Commission endorse efforts to monitor and address LGBTQ+ health disparities through the collection of sexual orientation and gender identity data [55], as well as Centers for Medicare and Medicaid Services (CMS) under President Biden [27]. The state of these data collection efforts, and more broadly LGBTQ+ patient protections, are precarious. In January 2025, President Trump signed multiple executive orders rescinding the previous administration's goals of developing inclusive demographic data, ending non‐discrimination protections for LGBTQ+ people, and restricting access to gender‐affirming care [56]. Health system leaders should ensure LGBTQ+ populations maintain adequate access to health services in the emergent policy landscape.

## 5 Conclusion

We used latent class analysis to identify variation in LGBTQ+ patient experiences. Most patients reported neutral experiences, a small portion of LGBTQ+ patients reported explicitly discriminatory experiences, and approximately one‐third of patients reported that their clinician had LGBTQ+ affirming practices. In the absence of these explicitly affirming practices, LGBTQ+ patients were less likely to receive recommended preventive services such as lifetime HIV testing, a recent HIV test, flu vaccination, and recent colorectal cancer screening. Multi‐level approaches to increase access to LGBTQ+ affirming care are needed to advance LGBTQ+ population health equity.

## Conflicts of Interest

The authors declare no conflicts of interest.

## Supporting information


**Data S1. Figure S1.** Distribution of responses to the original LCA indicators variables (not binarized).
**Table S1.** Healthcare Equality Index Scores of Healthcare Facilities in Q‐SNAPS sample states.

## Data Availability

The data that support the findings of this study are available from the corresponding author upon reasonable request.
