# Supplementary material for: Patterns of Lesbian, Gay, Bisexual, Transgender, and Queer Patient Experiences and Receipt of Preventive Services
Source: Health Serv Res. 2025 May 4;60(5):e14632. doi: 10.1111/1475-6773.14632 (PMC12461105; doi:10.1111/1475-6773.14632)
Supplement: Supplementary file 1 — Data S1. Figure S1. Distribution of responses to the original LCA indicators variables (not binarized). Table S1. Healthcare Equality Index Scores of Healthcare Facilities in Q‐SNAPS sample states. [file HESR-60-e14632-s001.docx]

**Appendix to** *Patterns of Lesbian, Gay, Bisexual, Transgender, and Queer Patient Experiences and Receipt of Preventive Services*

***Appendix Figure 1 Distribution of responses to the original LCA indicators* *variables (not binarized).***

Categorical variables with 5+ levels are often treated as a continuous variable in SEM. However, the mathematical assumption is that the data are normally distributed. Nonnormally distributed data are more likely to be biased (increase the AIC/BIC and reject appropriate models), as is the case for responses to the 3 negative healthcare experience indicators.

| ***Appendix Table 1. Healthcare Equality Index Scores of Healthcare Facilities in Q-SNAPS sample states*** | | | | | | |
| --- | --- | --- | --- | --- | --- | --- |
| State | Total | Independently Researched | Total Participated | Leaders | Top Performers | Participants |
| Georgia | 48 | 37 | 11 | 1 | 3 | 7 |
| North Carolina | 65 | 33 | 32 | 17 | 12 | 3 |
| Tennessee | 51 | 46 | 5 | 1 | 1 | 3 |
| Alabama | 35 | 31 | 4 | 1 | 3 | 0 |
| Healthcare Equality Index data accessed at <https://www.hrc.org/resources/healthcare-facilities/search/p2?q=georgia> | | | | | | |

*Assessing non-random missingness from Wave 1 to Wave 2.* Respondents who were lost to follow up at Wave 2 were more likely to report “*No, I don’t need or want*, *No, I cannot find*, *I don’t know,* or to skip the question about whether their providers’ clinical and cultural competence, or lack thereof, for working with LGBTQ+ patients.
